# Supplementary material for: Single cell RNA-seq reveals profound transcriptional similarity between Barrett’s oesophagus and oesophageal submucosal glands
Source: Nat Commun. 2018 Oct 15;9:4261. doi: 10.1038/s41467-018-06796-9 (PMC6189174; doi:10.1038/s41467-018-06796-9)
Supplement: Supplementary file 1 — Supplementary Information file [file 41467_2018_6796_MOESM1_ESM.pdf]

Single cell RNA-seq reveals profound transcriptional similarity between Barrett's oesophagus and oesophageal submucosal glands

Owen et al.

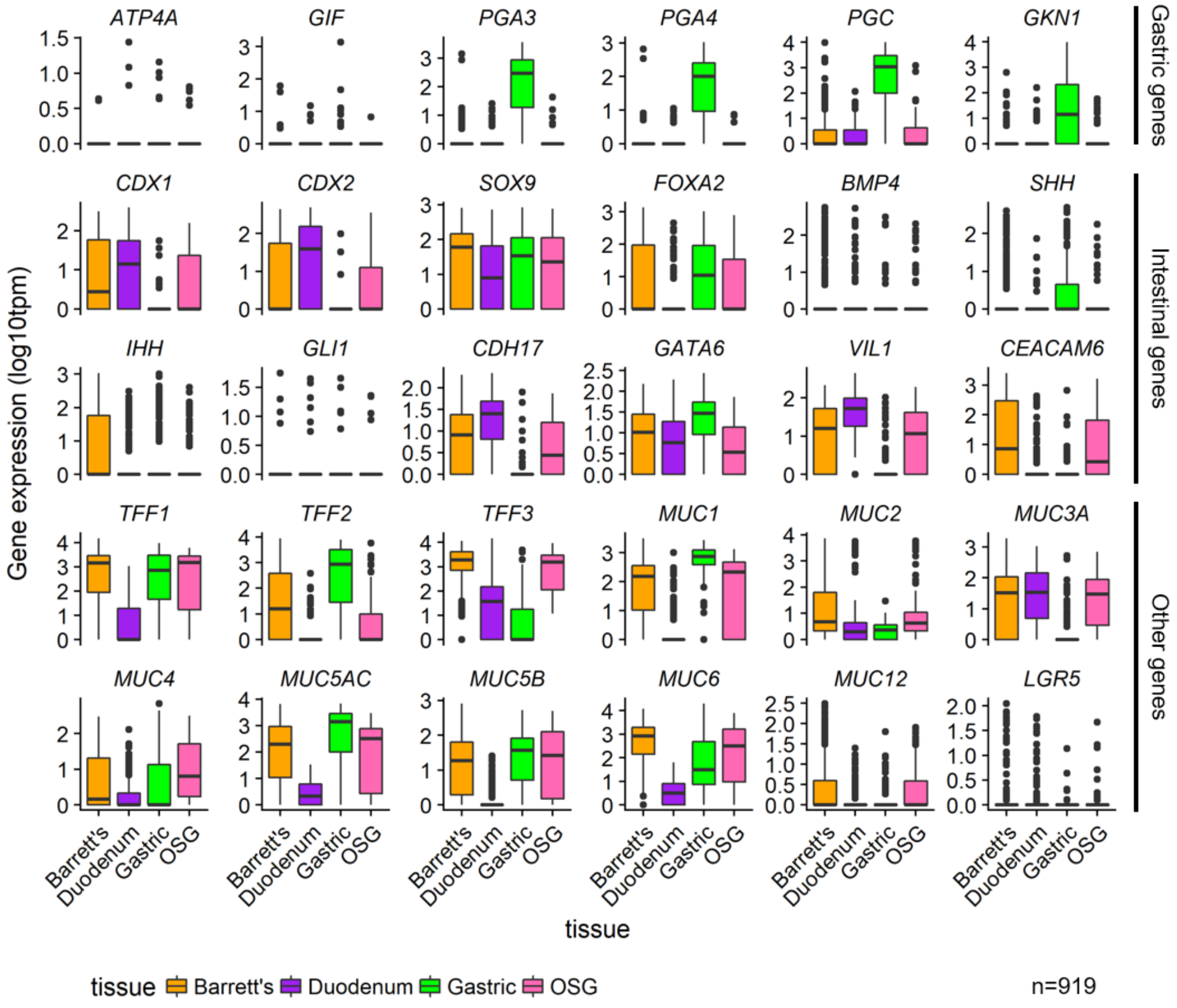

Supplementary Figure 1. Expression of selected tissue and cell defining genes in columnar cells

Boxplots showing the expression of a selection of genes used to define gastric or intestinal tissue cell types (first row of plots are gastric genes, second and third rows are intestinal genes, as indicated), and a panel of mucin and trefoil factor genes (fourth and fifth rows of plots). The lower and upper hinges of the boxplots correspond to the first and third quartiles (the 25th and 75th percentiles), the whiskers extend from the hinge to the largest or smallest values at most 1.5 x inter-quartile range from the hinge. Data beyond the whiskers are outliers and are plotted individually. Cells included are from all duodenum (n=207), gastric (n=221) and Barrett's samples (n=371), columnar type oesophageal cells are also included as 'OSGs' (n=120, as in Figure 3). Total n=919.

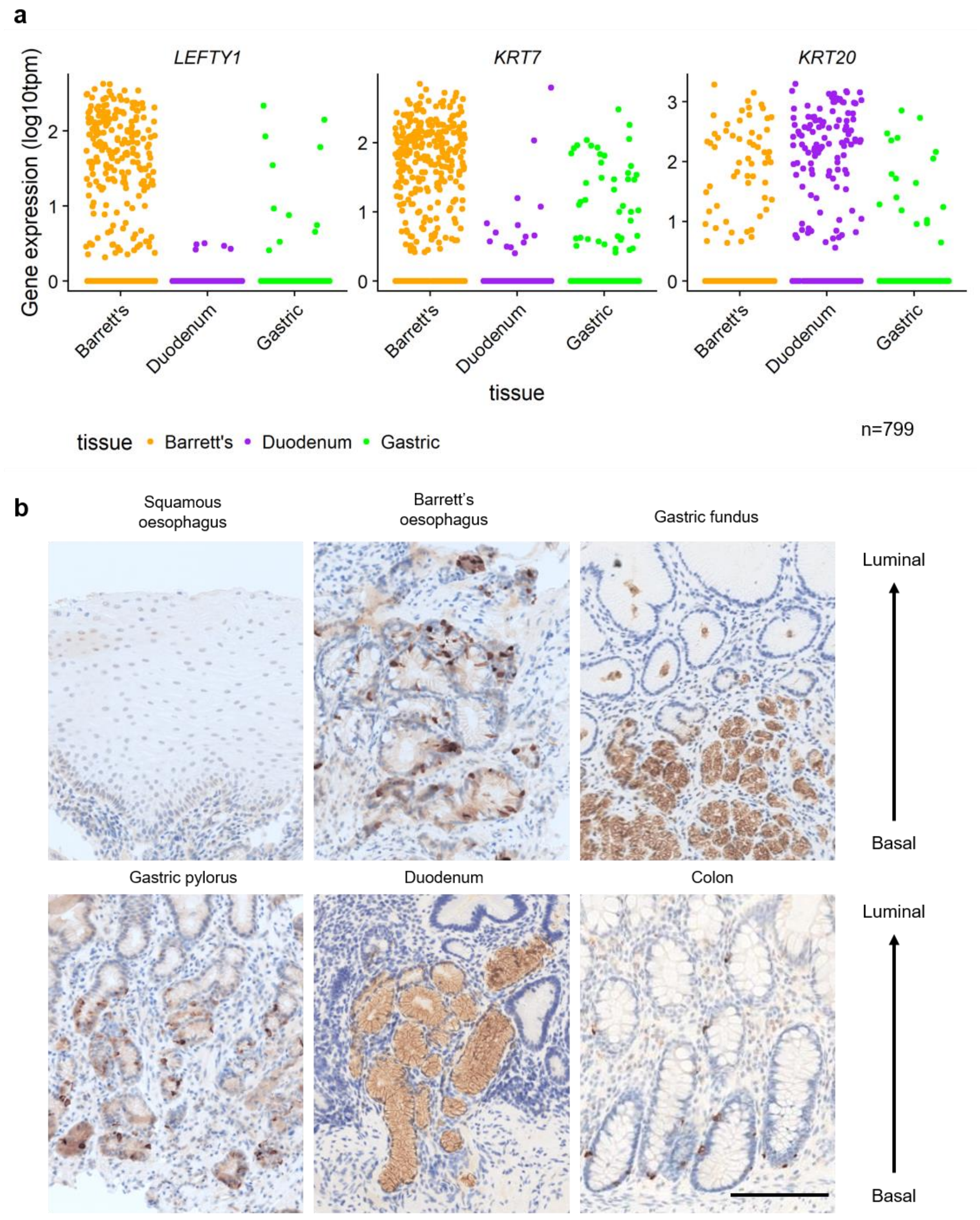

Supplementary Figure 2. Expression of *LEFTY1, KRT7* and *KRT20* in BO, duodenal and gastric cells

(a) Jitter plots showing the expression of *LEFTY1*, *KRT7* and *KRT20* in Barrett's, duodenum and gastric cells. **(b)** Immunohistochemical staining of LEFTY1 in normal tissues and Barrett's oesophagus as indicated. Scale bar 200μm.

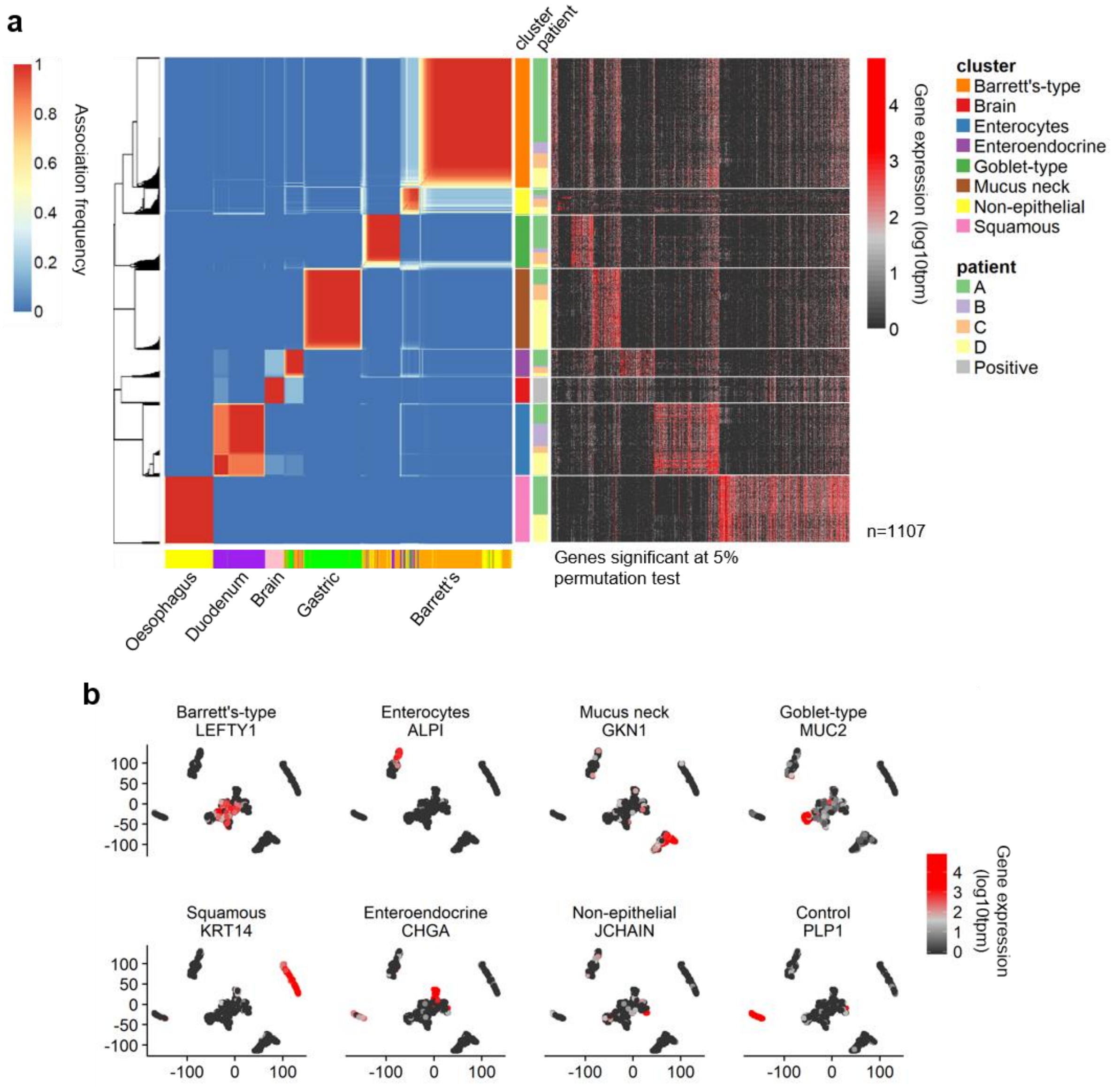

Supplementary Figure 3. Clustering and differential gene expression profiles of all single cells from Barrett's oesophagus patient samples

(a) The left heatmap shows a cluster consensus matrix for single cells from all tissue sites in BO patients (n=1107 including brain positive controls). Blue-to-red colours denote the frequency with which cells are grouped together in 250 repeat clusterings of simulated technical replicates (see Methods). Tissue type is indicated below. Cell clusters are labelled on the right with the cell type they contain or a descriptive term if that cell type has not been previously characterised. The right side heatmap shows differentially expressed genes (>4 fold,

genes significant at 5% permutation test) in each cluster from panel **a**. Cells from each patient are indicated by the coloured bar on the left. (**b)** t-SNE plots of all cells from BO patients (n=1107 including positive controls), coloured in each panel by transcript level of a gene that is highly expressed in a particular cluster type (cluster name and gene name shown above plots).

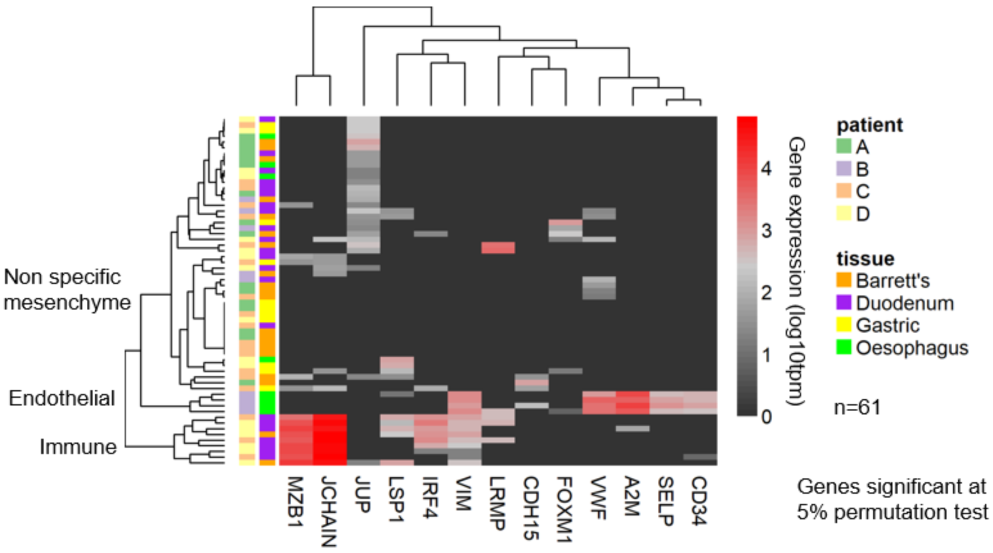

Supplementary Figure 4. Characterisation of 'non-epithelial' cells by gene expression

Heatmap of gene expression in cells in the cluster labelled as 'non-epithelial', showing genes significantly upregulated in this cluster (>4 fold, genes significant at 5% permutation test). Genes are clustered by nearest neighbour and the dendrogram is labelled by broad cell-type category based on known functions of the expressed genes. For each cell, the tissue type and patient is indicated by the coloured bars on the left.

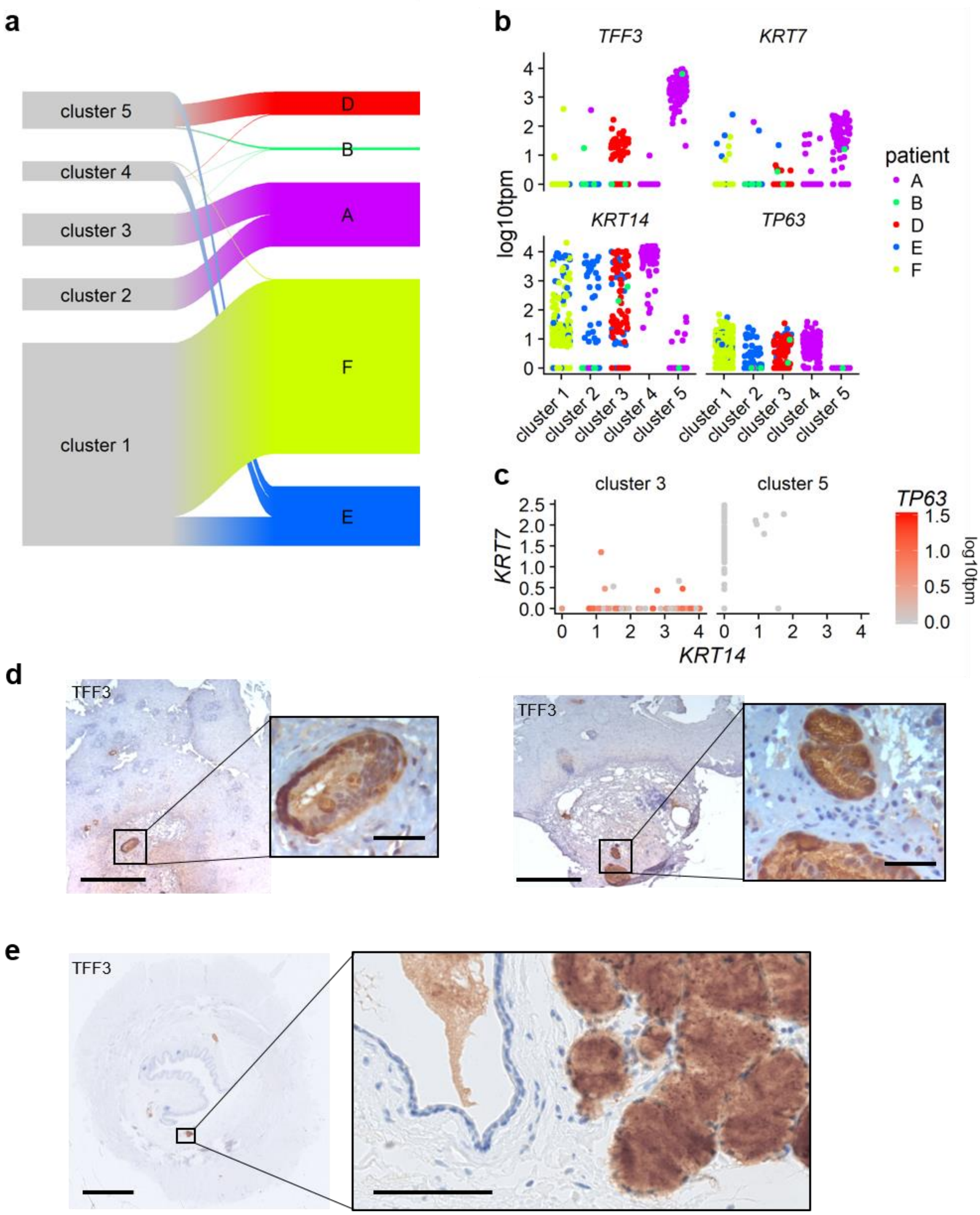

Supplementary Figure 5. Columnar gene-expressing cell detection in patients with and without Barrett's oesophagus

(a) Sankey diagram showing how oesophageal cells from patients with BO (A, B, D) and without (E and F) contribute to SC3 clusters generated using oesophageal cells only. **(b)** Jitter plots showing the expression of columnar (*TFF3* and *KRT7*) and squamous (*KRT14* and *TP63*)

genes in cells from **a** coloured by patient (log10tpm) across each cluster. **(c)** Scatter plots showing the expression of *KRT7*, *KRT14* and *TP63* in the two clusters which contain the most *TFF3*-expressing cells (units are log10tpm). **(d)** Immunohistochemical staining of TFF3 in OSGs from normal oesophageal samples obtained endoscopically from two Barrett's patients (scale bars 400µm), with enlarged images (scale bars 50µm). **(e)** Immunohistochemical staining of TFF3 in OSGs under normal oesophagus taken from the proximal part of an oesophagectomy specimen resected for Siewert type III junctional tumour in a patient with no BO. Scale bars are 1000µm and 100µm in enlarged images.

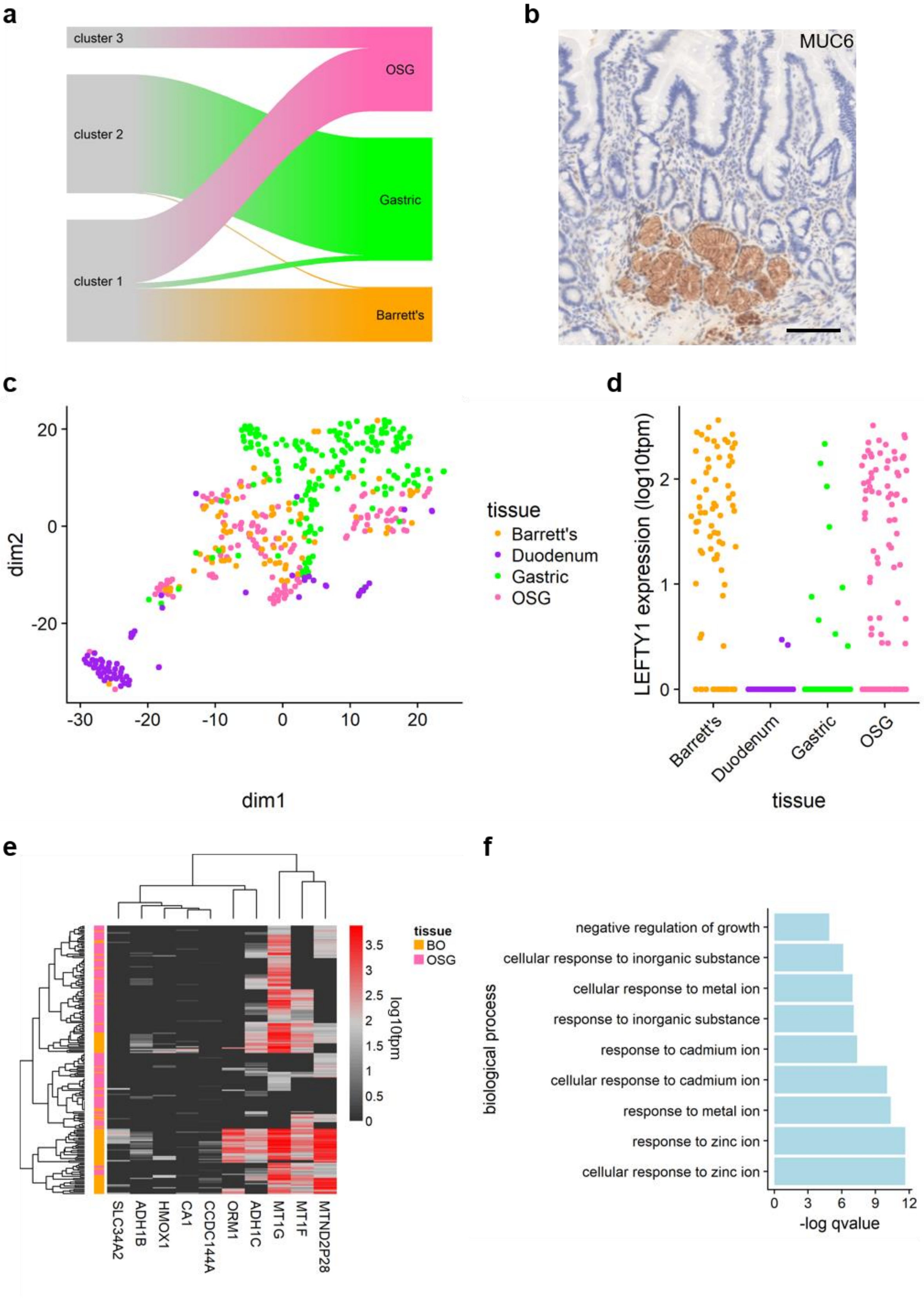

a
cluster 3
cluster 2
cluster 1
OSG
Gastric
Barrett's
b
MUC6
c
dim2
dim1
20
0
-20
-30
-20
-10
0
10
20
tissue
Barrett's
Duodenum
Gastric
OSG
d
LEFTY1 expression (log10tpm)
2
1
0
Barrett's
Duodenum
Gastric
OSG
tissue
e
tissue
BO
OSG
3.5
3
2.5
2
1.5
1
0.5
0
log10tpm
SLC34A2
ADH1B
HMOX1
CA1
CCDC144A
ORM1
ADH1C
MT1G
MT1F
MTND2P28
f
biological process
negative regulation of growth
cellular response to inorganic substance
cellular response to metal ion
response to inorganic substance
response to cadmium ion
cellular response to cadmium ion
response to metal ion
response to zinc ion
cellular response to zinc ion
0
3
6
9
12
-log qvalue

Supplementary Figure 6. The majority of Barrett's oesophagus cells have a similar transcript profile to oesophageal submucosal gland (OSG) cells

(a) Sankey diagram showing how gastric, oesophageal and BO cells contribute to SC3 clusters of 'gland-like' cells from these tissues, which are a sub-set of gastric (n=175), BO (n=78) and oesophagus cells (n=120): excluding gastric and BO cells that expressed *CHGA* or *MUC2* (to exclude enteroendocrine and goblet cells, respectively), and excluding oesophageal cells that did not express *TFF3* (to exclude squamous cells). Thresholds were set at the tenth centile of cells in which at least one transcript was detected from each gene of interest. **(b)** Immunohistochemistry of MUC6 in duodenum showing positive expression in a Brunner's gland. Scale bar 200μm. **(c)** t-SNE plot of 'gland-like' cells as in **a** but also including duodenal cells expressing maximal *MUC6* (to enrich for Brunner's gland type cells, n=65). The threshold for including a duodenal cell was set at the fiftieth centile of *MUC6* expression. **(d)** Jitter plot showing expression of *LEFTY1* in 'gland-like' cells as in **c** (n=438). **(e)** Heat map with hierarchical clustering of genes and samples showing the top ten most differentially upregulated genes between BO cells (n=78) and oesophagus cells (n=120): excluding gastric and BO cells that expressed *CHGA* or *MUC2* (to exclude enteroendocrine and goblet cells, respectively), and excluding oesophageal cells that did not express *TFF3* (to exclude squamous cells). **(f)** Enriched gene pathways (FDR < 0.01), based on the full results of **e**.

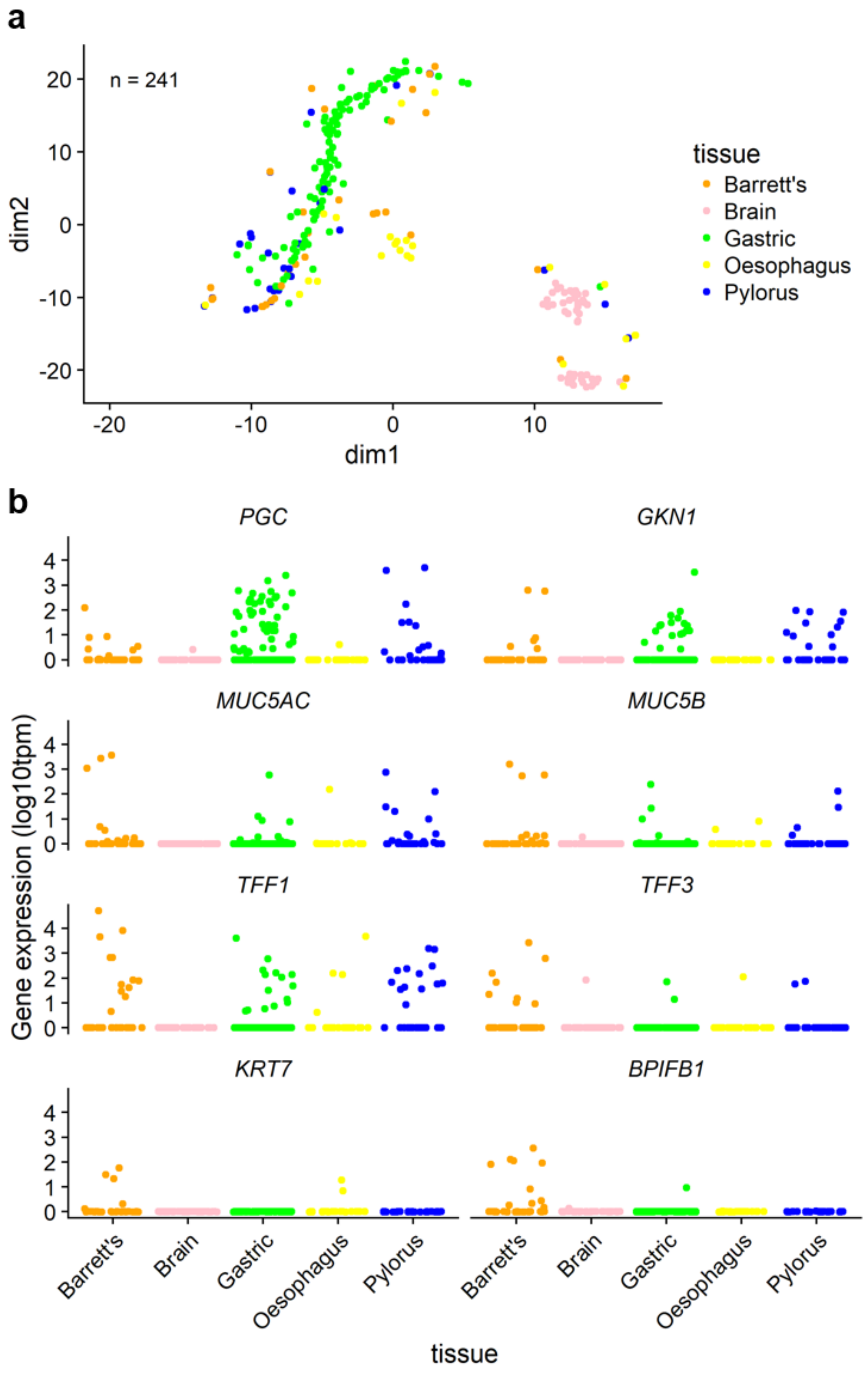

Supplementary Figure 7. Cells from gastric cardia and pylorus have similar RNA compositions.

(a) t-SNE plot of gastric cardia cells (n=109), gastric pylorus cells (n=29), BO cells (n=32), oesophagus cells (n=24) and brain RNA control (n=47) showing transcriptional relationships

between cells obtained from an additional two patients with BO. **(b)** Jitter plots showing expression of selected epithelial genes in gastric cardia, gastric pylorus, BO and oesophagus cells as in **a**.

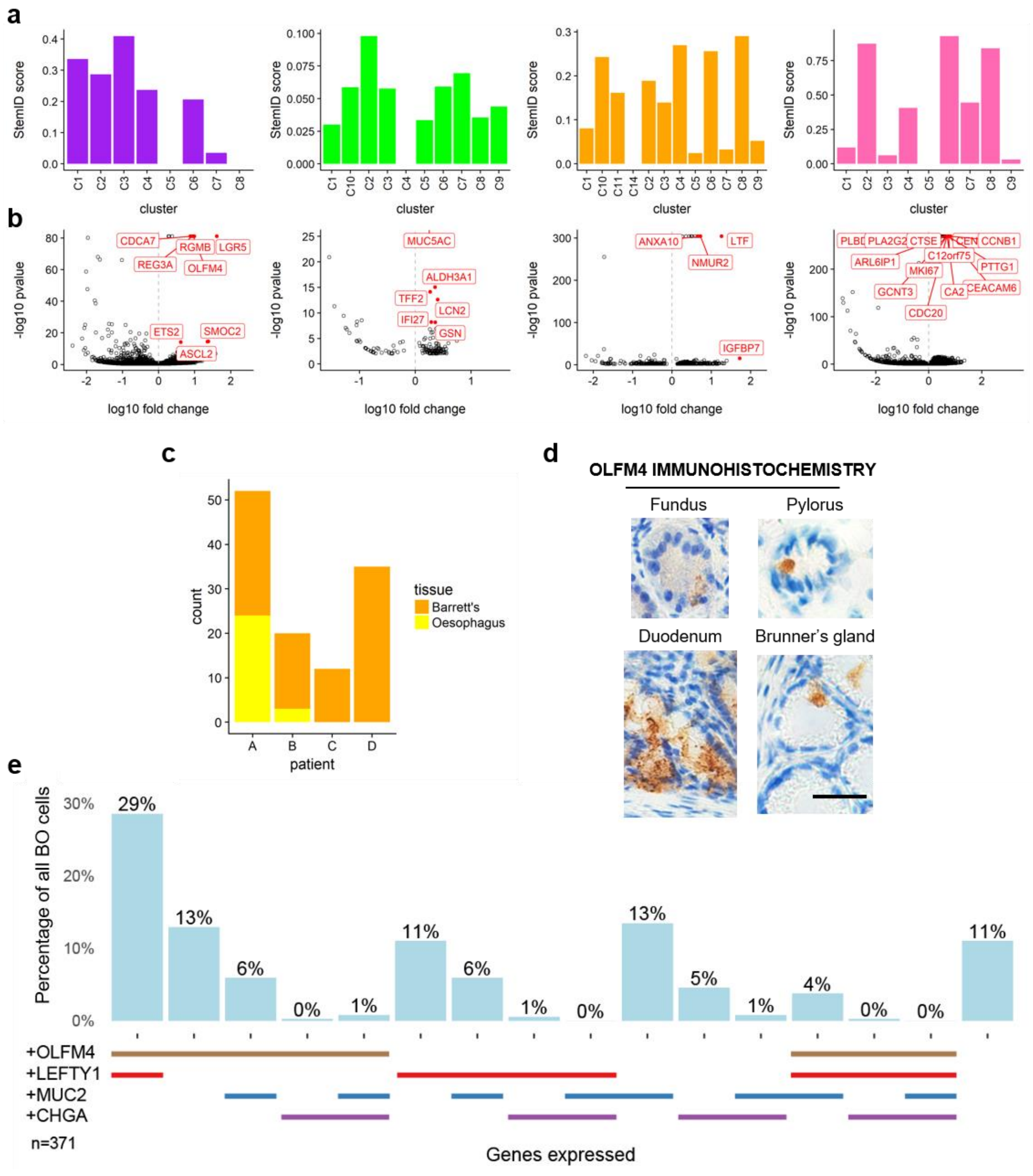

Supplementary Figure 8. StemID applied to human GI cells with expression of stem and associated markers in BO.

(a) StemID scores across all clusters computed for duodenum (purple), gastric (green), Barrett's oesophagus (orange) and oesophageal submucosal gland cells (pink) in bar plots from left to right. Scores are calculated from multiplication of the entropy (spread from the cluster mean) and the number of cluster links arising from a given cluster. Oesophageal submucosal

gland cells were deduced by removing squamous (*KRT14+*) cells from all normal oesophagus cells from patients A-D (cells from patients E and F were removed to avoid comparing cells between batches). **(b)** Volcano plots showing significantly expressed genes in the highest scoring cluster corresponding the plot directly above in **a**, from left to right these are duodenum, gastric, Barrett's oesophagus and oesophageal submucosal gland cells. **(c)** Number of Barrett's and OSG cells isolated from each patient and represented in the highest scoring StemID cluster in **Figure 5** (cluster 3). **(d)** Immunohistochemical staining of OLFM4 in control tissues and structures as indicated. Scale bars are 50µm. **(e)** Single cell RNA-seq data showing percentage of cells expressing *OLFM4*, *LEFTY1*, *MUC2* or *CHGA* alone and in all combinations (thresholds for calling a gene 'expressed' were set at the tenth centile to include 90% of cells in which at least one transcript was detected from each gene).

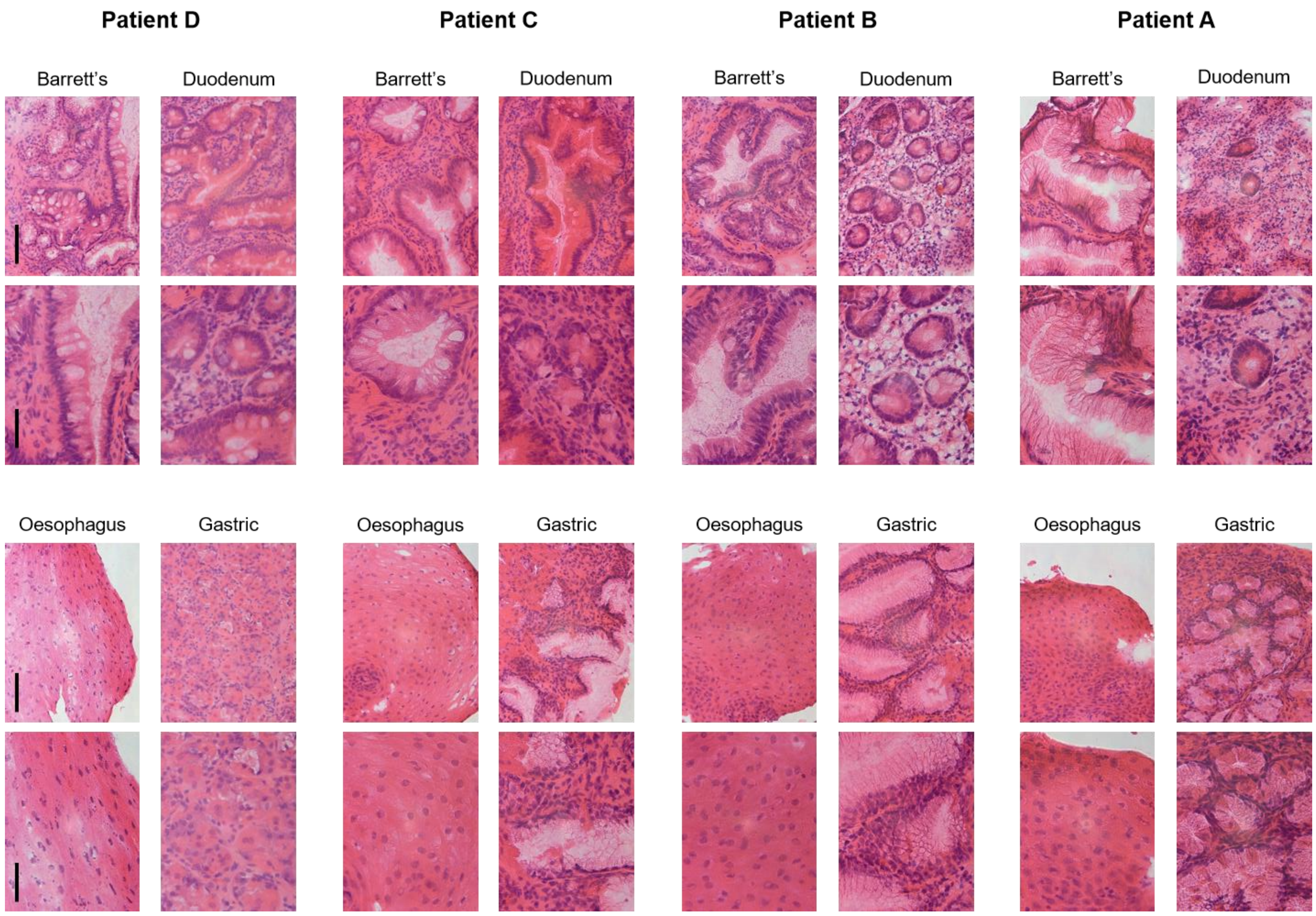

Patient D
Patient C
Patient B
Patient A
Barrett's
Duodenum
Barrett's
Duodenum
Barrett's
Duodenum
Barrett's
Duodenum
Oesophagus
Gastric
Oesophagus
Gastric
Oesophagus
Gastric
Oesophagus
Gastric

Supplementary Figure 9. Histology of Barrett's patient biopsies used for single cell analysis.

Haematoxylin and eosin staining of frozen sections taken from the biopsy fragments that were used for single cell and bulk RNA-sequencing from four patients (excluding the normal oesophageal samples taken from non-Barrett's oesophagus patients). Images on the right of each pair are a higher magnification of the image on the left. Each scale bar (on lower-most images) applies to the whole column; 100µm in first and third columns, and 50µm for the second and fourth columns.

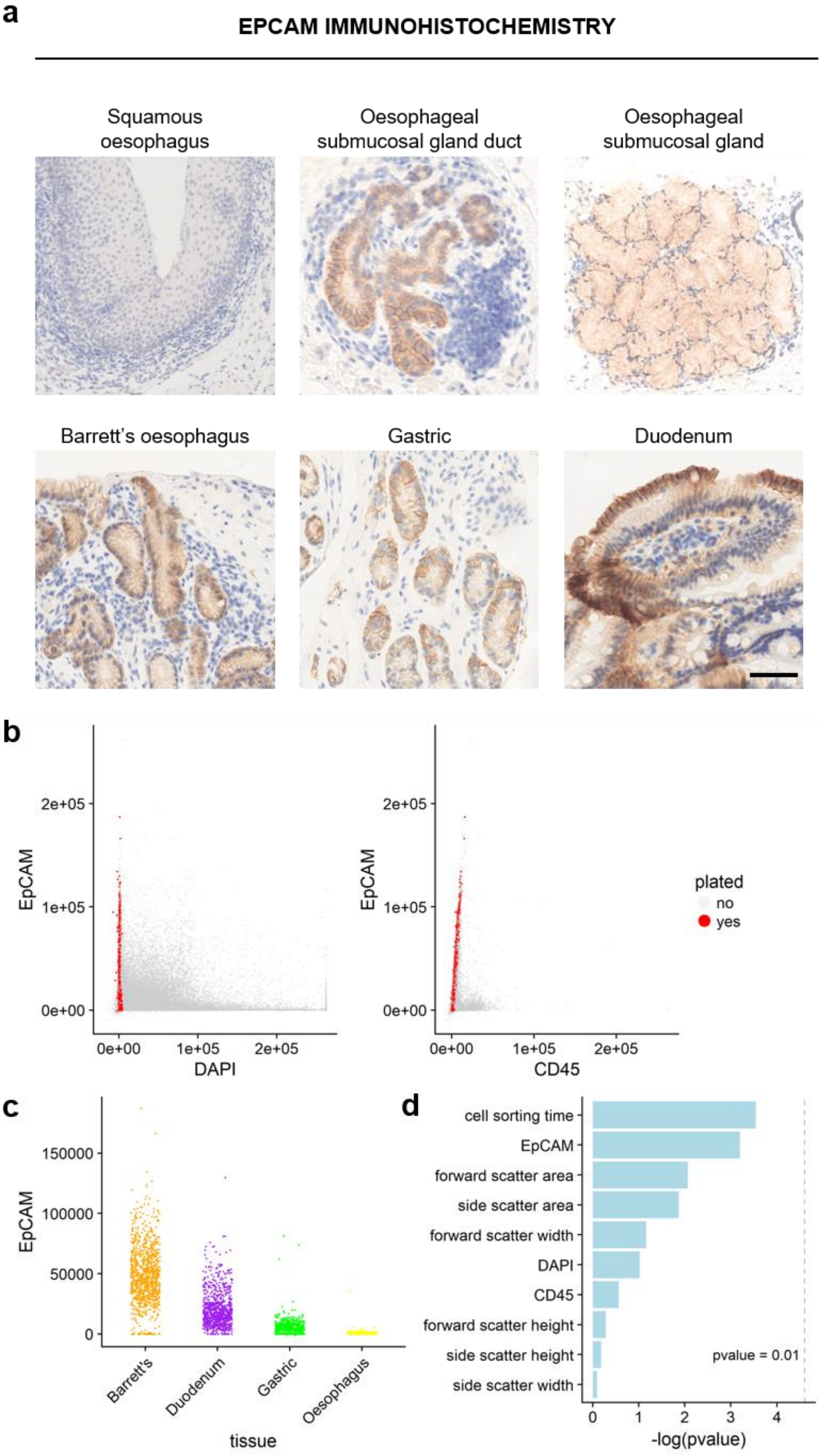
a
EPCAM IMMUNOHISTOCHEMISTRY
Squamous oesophagus
Oesophageal submucosal gland duct
Oesophageal submucosal gland
Barrett's oesophagus
Gastric
Duodenum
b
EpCAM
DAPI
CD45
plated
no
yes
c
EpCAM
tissue
Barrett's
Duodenum
Gastric
Oesophagus
d
cell sorting time
EpCAM
forward scatter area
side scatter area
forward scatter width
DAPI
CD45
forward scatter height
side scatter height
side scatter width
pvalue = 0.01
-log(pvalue)

Supplementary Figure 10. Fluorescence activated cell sorting shows characteristics of cells selected for single cell RNA-sequencing.

(a) Immunohistochemical staining of EpCAM in oesophageal, Barrett's, gastric and duodenal epithelium. Scale bar 100µm. **(b)** Scatter plots showing the expression levels of the white cell marker CD45, the epithelial marker EpCAM, and the cell viability marker DAPI (high expression indicates non-viable cell) in all fluorescence activated cell sorting events, separated by cells which were sorted into 96 well plates (red), and those which were not (light grey). **(c)** Jitter plot showing the expression levels of EpCAM in plated duodenum, gastric, BO and oesophagus cells (coloured red in **b**) which passed the threshold for further analysis with those that did not. **(d)** Logistic regression comparing plated cells (coloured red in **b**) which passed the threshold for further analysis, set with positive and negative controls, with those that did not. Vertical black dotted line indicates p value threshold of 0.01, which none of the predictors exceed.

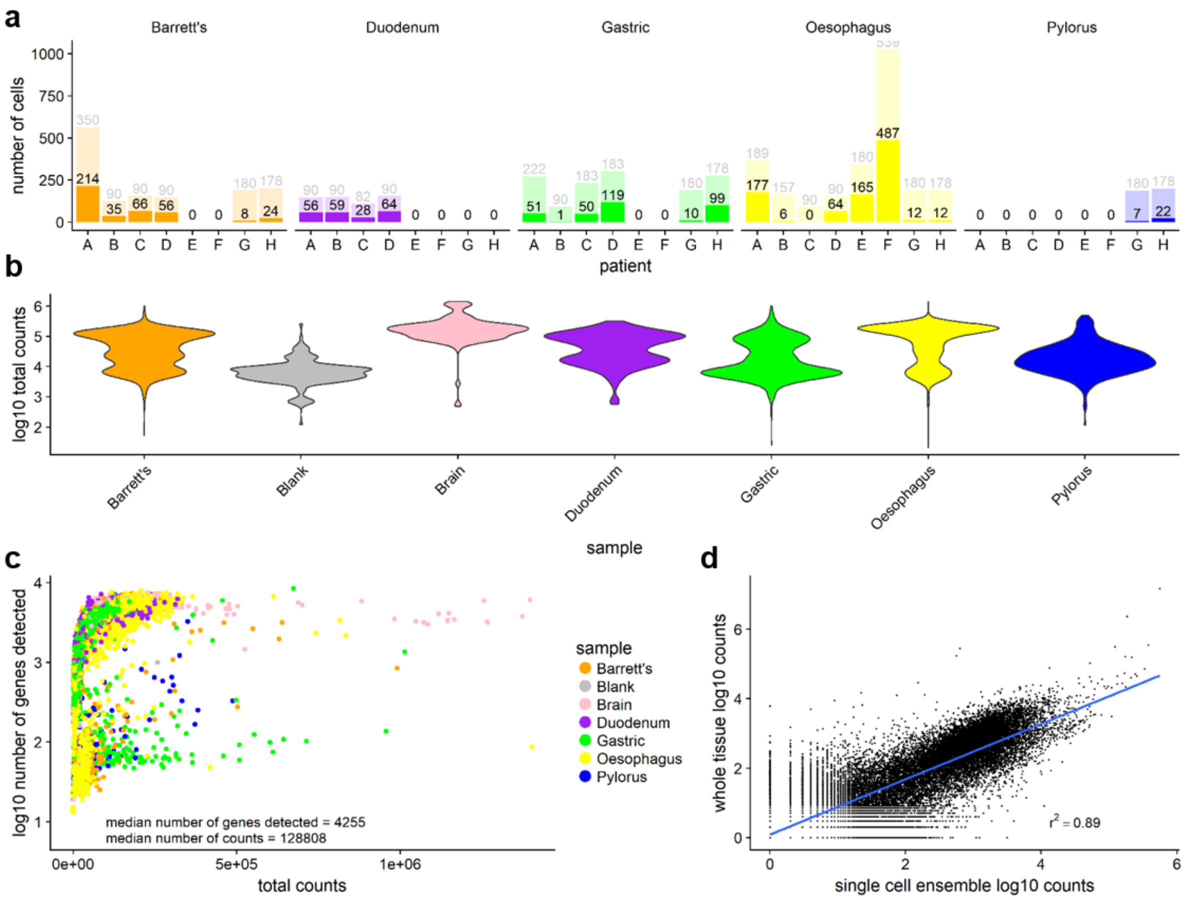

Supplementary Figure 11. Sample analysis and quality control

(a) Bar plot showing total number of cells sequenced of each tissue type by patient (A-D and G-H are patients with BO, E-F are patients without BO). Cells passing threshold for inclusion determined by controls (see **Methods**) are in solid colour, excluded cells are shown in faded colour. **(b)** Violin plots showing number of counts by each tissue type, coloured as in **a**, in all cells which were sequenced. **(c)** Plot of number of genes detected (at least one count per gene) against total counts for every sequenced cell and control. **(d)** Scatter plot showing the correlation between bulk tissue RNA-seq gene counts and an ensemble of single cell gene counts, taken from Barrett's samples from patient A, with Pearson correlation coefficient displayed.

Patient	Age	Sex	Smoking (cigs/day)	Alcohol (units/week)	BMI	Prague	scRNA-seq	Bulk RNA-seq
A	78	M	0	21	26.2	C3M5	Yes	Yes
B	77	M	0	4	25.7	C0M3	Yes	Yes
C	47	F	0	16	22.2	C0M2	Yes	Yes
D	90	F	0	6	23.7	C9M11	Yes	Yes
E	82	F	0	0	27.1	na	Yes	No
F	68	F	0	0	25.8	na	Yes	No
G	76	M	10	8	27.5	C1M3	Yes	No
H	78	M	0	22	29.4	C2M4	Yes	No
I	87	M	ex 10/day	8	21.9	C8M10	No	Yes
J	75	F	0	2	35.7	C1M3	No	Yes
K	74	M	ex 1/day	24	25.5	C2M3	No	Yes
L	63	M	ex 15/day	10	22.8	C0M3	No	Yes
M	70	F	0	4	32.6	C5M7	No	Yes
N	59	M	0	15	41.0	C0M1	No	Yes
O	67	F	0	0	31.7	C13M15	No	Yes
P	65	F	30/day	3	27.3	C0M1	No	Yes
Q	49	M	20/day	0	42.9	C3M4	No	Yes

Prague score: C = Cirumferential extent of lesion; M = Maximal extend of lesion

Supplementary Table 1. Details of patients' samples having single cell and bulk RNA-sequencing.

BMI, body mass index; PPI, protein pump inhibitor treatment. Prague indicates Prague classification for measuring the length of Barrett's oesophagus, where C is proximal extent of circumferential lesion and M is maximal proximal extent (na for patients without BO). scRNA-seq and Bulk RNA-seq indicate whether samples from each patient were used for single cell or bulk RNA-seq, respectively, total n=17.

Patient sample #	Histological diagnosis	Number of sections stained for ITLN1/SPINK4/MUC2
8173	BE with IM	3
8226	BE with IM	3
5368	BE with IM some LGD	3
6198	BE with IM some LGD	3
8720	BE with IM	3
452	BE with IM	3
3458	BE with IM	2
9811	BE with IM	2
5097	BE with IM some LGD	1
6773	BE with IM some LGD	1
3612	BE with IM some LGD	1
7974	BE with IM	1
6446	BE with IM some LGD	1
813	BE with IM some LGD	1
2318	BE with IM	1
7709	BE with IM some LGD and HGD	1

Supplementary Table 2. Summary of immunofluorescence staining of BO specimens.

Integers denote the number of sections (one section per slide) triple stained for ITLN1, MUC2 and SPINK4. A total of 30 specimens were stained from 16 patients (a different cohort to the patients in Extended Data Table 1). Basic pathological details are noted (BO, Barrett's oesophagus; IM, intestinal metaplasia; LGD, low grade dysplasia; HGD, high grade dysplasia).

Antibody			Patient sample #									
			8173		8226		5097		6446		5073	
MUC2	SPINK4	ITLN1	n	%	n	%	n	%	n	%	n	%
High	High	High	22	6	34	12	41	16	15	5	2	3
High	High	Low	28	7	26	9	6	2	190	59	4	5
High	Low	High	11	3	17	6	4	2	2	1	3	4
High	Low	Low	150	38	54	18	30	12	55	17	44	55
Low	High	High	40	10	134	45	70	28	26	8	11	14
Low	High	Low	110	28	11	4	7	3	20	6	4	5
Low	Low	High	16	4	7	2	90	36	11	3	7	9
Low	Low	Low	17	4	12	4	5	2	5	2	5	6
		Total cells	394		295		253		324		80	
MUC2 Low, SPINK4 and/or ITLN1 High			166	42	152	52	167	66	57	18	22	28
MUC2 High, SPINK4 and ITLN1 Low			150	38	54	18	30	12	55	17	44	55

Supplementary Table 3. Quantification of MUC2, ITLN1 and SPINK4 immunofluorescence triple-staining in BO.

Table summarising MUC2, ITLN1 and SPINK4 immunofluorescence triple-stain expression pattern in sections of BO from 5 patients, sections contained BO with intestinal metaplasia but no dysplasia and no carcinoma. n is the number of cells scored as positive for expression of each protein by immunofluorescence. % is the percentage of the total number of scored cells (bottom row) that is positive for the protein(s) listed. See **Methods** for details of the antibodies used.
