# Supplementary material for: Single cell RNA-seq reveals profound transcriptional similarity between Barrett’s oesophagus and oesophageal submucosal glands
Source: Nat Commun. 2018 Oct 15;9:4261. doi: 10.1038/s41467-018-06796-9 (PMC6189174; doi:10.1038/s41467-018-06796-9)
Supplement: Supplementary file 10 — Description of Additional Supplementary Files [file 41467_2018_6796_MOESM10_ESM.docx]

**Title:** Supplementary Data 1.

**Description:** Differentially expressed genes between tissue-specific cell clusters. Tables containing differentially expressed genes between clusters for each of the Barrett’s oesophagus, duodenum, gastric and oesophagus cells in patients A-D. Differentially expressed genes were obtained by comparing cells of each cluster against all other clusters in a given tissue type. See Methods for details on how clusters were determined, and thresholds for significance were determined.

**Title:** Supplementary Data 2.

**Description:** Summary of immunohistochemical staining of BO specimens. Table summarising the pathology and immunohistochemical staining results of patients with BO and control sites. A total of 140 Barrett’s biopsies from 80 patients were immunohistochemically stained for LEFTY1, OLFM4, TFF3, MUC2, CK7, KI67, CHGA. Biopsies are characterised by one of non-goblet columnar gland, Barrett’s gland, fundic gland, oxynto-cardiac glands (none of the samples exhibited Paneth cells). 31 endoscopic biopsies from control sites of 26 BO patients were also stained. An additional 5 pyloric biopsies, 5 oesophageal resection specimens and 5 normal colon samples were stained for the same markers.

**Title:** Supplementary Data 3.

**Description:** Differentially expressed genes between all tissue clusters. Tables containing differentially expressed genes between clusters for all cells analysed in patients AD. Differentially expressed genes were obtained by comparing cells of each cluster against all other clusters. See Methods for details on how clusters were determined, and thresholds for significance were determined.

**Title:** Supplementary Data 4.

**Description:** Differentially expressed genes between high scoring StemID determined cell clusters. Tables containing all genes analysed using StemID (see Methods for details). Tables show the differential expression of genes in clusters with high StemID scores (as seen in the plots in Figure 5 and Supplementary Fig. 8) in BO, OSGs, duodenum, gastric and combined BO and OSG cells.

**Title:** Supplementary Data 5.

**Description:** Gene counts for all bulk samples. A table containing processed gene counts for all bulk tissue samples sequenced (see Methods for details of how gene counts were determined from raw RNA-seq data).

**Title:** Supplementary Data 6.

**Description:** Gene counts for all cells. A table containing processed gene counts for all cells sequenced (see Methods for details of how gene counts were determined from raw RNA-seq data).

**Title:** Supplementary Data 7.

**Description:** Cell and clustering metadata. A table containing all cells (as in Supplementary Data 5), each with details about which patient and tissue they were derived from, as well as which cluster each cell belongs to in each analysis performed, where this is applicable.
